# CEBPB promotes ulcerative colitis-associated colorectal cancer by stimulating tumor growth and activating the NF-κB/STAT3 signaling pathway

**DOI:** 10.1515/biol-2022-1012

**Published:** 2025-06-06

**Authors:** Shan Gao, Wei Wang, Xu-Dong Tong, Dao-Rong Wang, Ai-Xia Tian, Wei Yang, Jin-Min Chen

**Affiliations:** Department of Gastroenterology, Xiangyang Central Hospital, Affiliated Hospital of Hubei University of Arts and Science, No. 136, Jingzhoue Street, Xiangyang, 441021, Hubei, China

**Keywords:** colorectal cancer, CEBPB, ulcerative colitis, NF-κB/STAT3

## Abstract

Ulcerative colitis-associated colorectal cancer (UCCRC) represents a significant complication of ulcerative colitis. CEBPB has been shown to promote the invasion of colon cancer cells. In this study, we aimed to investigate the role of CEBPB in the progression of cancers associated with colitis. The wild-type (WT) mice, transfected with a vector expressing CEBPB and siRNA targeting CEBPB, along with their littermate controls, were subjected to a challenge using azoxymethane and dextran sodium sulfate to establish a model of UCCRC. Colon tissues and blood samples were collected for analysis through hematoxylin and eosin staining and enzyme linked immunosorbent assay. Immunohistochemical staining was employed to assess protein expression. In the UCCRC model, mice transfected with vectors expressing CEBPB exhibited a reduction in weight loss and colorectal stenosis, as well as disordered colonic gland structure. Additionally, these mice demonstrated an increased number and size of tumors compared to WT controls. Furthermore, transfection with CEBPB resulted in a decrease in both the quantity and dimensions of tumors. NF-κB was found to enhance the phosphorylation level of STAT3 based on Western blot assay. The activation of NF-κB and STAT3 subsequently promoted the proliferation, invasion, and migration of colon cancer cells by clone formation assays, transwell assays, and scratch-wound assays. Moreover, rescue experiments indicated that CEBPB induced UCCRC through the NF-κB/STAT3 signaling pathway. CEBPB mediated colonic injury in UCCRC mice by activating the NF-κB/STAT3 pathway. This finding reveals a previously unrecognized link between CEBPB and colitis-related tumorigenesis and provides new insight into UCCRC pathogenesis.

## 1 Introduction

Ulcerative colitis (UC) is a chronic inflammatory condition that primarily impacts the mucosal and submucosal layers of the large intestine. It is characterized by symptoms such as abdominal pain, hematochezia, and diarrhea [1]. The severity of UC can vary and may recur over time. Unfortunately, there is currently no universally accepted standard for the diagnosis of UC. Diagnosis typically involves a comprehensive approach that includes clinical presentation, biochemical analysis, fecal examination, endoscopy, imaging studies, and pathological findings [2]. Patients with UC face an increased risk of developing colon cancer, which represents a significant complication and a leading cause of mortality in cases of ulcerative colitis [3]. Furthermore, the severity of colon inflammation serves as an independent risk factor for the emergence of colon neoplasms in individuals with chronic UC.

Currently, various signaling pathways and pivotal molecular nodes are associated with colitis and colon cancer. Research has demonstrated that oxidative stress induced by nitrogen and oxygen compounds within the inflammatory microenvironment, coupled with the activation of NF-κB and STAT3 signaling pathways by inflammatory mediators, plays a crucial role in the progression from colitis to colon cancer [4,5]. Persistent activation of the NF-κB signaling pathway can result in DNA damage to intestinal epithelial cells and expedite the development of intestinal adenomas [6,7]. Mutant p53 has the capacity to prolong this activation, leading to chronic inflammation and contributing to the emergence of inflammation-related colorectal cancer [8,9]. Additionally, the activation of the interleukin (IL)-6-STAT3 signaling pathway can facilitate the progression of colonic inflammation to cancer [10].

CEBPB is a member of the CCAAT family of element-binding proteins, consisting of six basic leucine zipper DNA-binding proteins. It functions as a homodimer to specifically bind to regulatory regions within DNA [10,11]. CEBPB plays a crucial role in regulating inflammatory genes and facilitating mitosis across various cell types. It was found that CEBPB is upregulated in samples of ulcerative colitis-associated colorectal cancer (UCCRC) and constitutes part of an NF-κB-related gene signature [12]. Additionally, CEBPB regulates gene transcription in response to the expression of IL-1 and IL-6 by activating the NF-κB and STAT pathways [13]. This modulation can exacerbate the inflammatory microenvironment, which is critical to the cancer microenvironment. Chronic inflammation has been associated with malignant cell transformation and the onset of cancer. Therefore, this study aims to investigate how the expression of CEBPB may regulate the occurrence and progression of UCCRC.

## 2 Methods

### 2.1 Mice and UCCRC model

About 90 male wild-type C57BL/6 mice was obtained from Heyuan Biotechnology Co., LTD (SYXK(HU)2017-0003), which were 8–10 weeks old and weighed 20 ± 1.2 g. To ensure their health and safety, they were kept in a specific pathogen-free (SPF) environment. All *in vivo* procedures were conducted in compliance with the Animal Experiment Administration Committee of the university. To induce UCCRC, which was previously described in a study, mice were injected with azoxymethane (AOM) (12 mg/kg) intraperitoneally. After 7 days, they were administered 2% dextran sodium sulfate (DSS) dissolved in drinking water for 7 consecutive days, followed by 14 days of regular drinking water for recovery. This cycle was repeated twice, followed by regular drinking water until day 84 when the mice were euthanized. Littermate controls were injected with normal saline (NS, 12 mg/kg) intraperitoneally, and the procedure was the same as mentioned earlier. Recombinant adeno-associated virus serotype 9 (rAAV9) expressing a siRNA targeting CEBPB (siCEBPB), full-length CEBPB cDNA (CEBPB), and scramble control (NC) were generated by Biowit Technologies Co., Ltd. (Shenzhen, China), according to the manufacturer’s protocol. In the CEBPB-treated groups, CEBPB or siCEBPB was tail vein-injected at a dose of 1 × 10^11^ vg on days 1, 3, and 5 during DSS treatment. In IkappaB kinase-2 (IKK2) inhibitor 2-[(aminocarbonyl)amino]-5-(4-fluorophenyl)-3-thiophenecarboxamide (TPCA-1)-treated groups, TPCA-1 (10 mg/mL; Tocris Bioscience, Ellisville, Missouri) was intraperitoneally injected from days 1 to 56 during DSS treatment. The colons were collected and cut open longitudinally to measure the tumor numbers and sizes. Simultaneously, the body weight and disease activity index (DAI) of the mice were recorded. Eventually, the mice were euthanized, placed in a plexiglass chamber with 5% isoflurane for 5 min, and decapitated when sufficiently sedated. Colorectal length and associated indicators were measured, and the colon tissues were either frozen in liquid nitrogen or fixed with formaldehyde (4%) and embedded in paraffin for downstream assays.


**Ethical approval:** The research related to animal use has been complied with all the relevant national regulations and institutional policies for the care and use of animals, and has been approved by the Ethics Committee of Animal Experiments of Hubei University of Arts and Science.

### 2.2 Hematoxylin and eosin (H&E) staining for mice’s colon tissues

After the paraffin-embedded colon tissues of mice were cut into sections, they underwent a series of treatments to prepare them for observation under a light microscope. Xylene and ethanol were used to remove any remaining wax from the tissue samples, ensuring that they were clean and ready for staining. The Hematoxylin and Eosin Staining Kit (Beyotime Biotechnology Co., LTD, Shanghai, China) was then applied according to the manufacturer’s instructions, which allowed for clear visualization of the colon tissues. This process is essential in order to accurately analyze any changes or abnormalities present in the tissue samples. By observing these tissues under a microscope, researchers can gain valuable insights into various diseases and conditions affecting the colon.

### 2.3 Determination of the concentrations of tumor necrosis factor alpha (TNF-α), IL-6, and IL-1β

After the harvest of mice’s blood, the sera were carefully separated to ensure accurate results. The enzyme linked immunosorbent assay (ELISA) kits from Beyotime were used to determine the serum concentrations of TNF-α, IL-6, and IL-1β. This method is widely recognized as a reliable way to measure cytokine levels in biological samples.

The absorbance at a wavelength of 450 nm was measured according to the manufacturer’s protocol (Beyotime). This allowed for precise quantification of each cytokine present in the serum samples. These measurements are important because they provide insight into how different immune cells respond during an inflammatory response.

### 2.4 Immunohistochemical (IHC) analysis

After the initial preparation steps of deparaffinization and rehydration, the sections underwent antigen retrieval with 1% sodium citrate to enhance the detection of specific proteins. To prevent interference from endogenous catalase, a 3% H_2_O_2_ solution was applied for 30 min to block its activity. The next step involved treating the sections with a 3% bovine serum albumin solution (Beyotime) to minimize non-specific binding of antibodies. Primary antibodies against E-catenin and Ki67 were then added and allowed to incubate overnight at 4°C. This process is crucial in identifying cellular markers that can provide insight into various biological processes such as cell proliferation and adhesion. By carefully following these procedures, researchers can obtain reliable results that contribute to our understanding of complex biological systems. After being washed with phosphate buffer saline (PBS) thrice to remove any residual debris or contaminants, the sections were carefully incubated with HRP (horseradish peroxidase; Beyotime)-conjugated secondary antibody for 1 h at room temperature (RT). This step is crucial in order to ensure that the primary antibody has bound specifically to its target antigen. The use of a conjugated secondary antibody allows for easy detection and visualization of the primary antibody–antigen complex.

Once the sections have been incubated with the secondary antibody, they are developed using 3,3′-diaminobenzidine (DAB; Beyotime) chromogenic solution. DAB reacts with HRP to produce a brown precipitate at sites where the primary antibody has bound to its target antigen. This reaction can be monitored under a light microscope and provides valuable information about protein expression patterns within tissues. To counterstain the nuclei and provide contrast against the brown precipitate, hematoxylin is applied after development. Hematoxylin stains DNA-rich regions blue-purple, allowing for clear visualization of cell nuclei within tissue sections.

Finally, coverslips are added using mounting medium in order to protect and preserve the stained tissue sections. The slides can then be visualized under a light microscope at various magnifications depending on experimental needs. Overall, this protocol provides an effective method for detecting specific proteins within tissue samples through immunohistochemistry techniques. The immunoreactive score (IRS) is a widely used method for evaluating the expression of proteins in tissue samples. It takes into account both the percentage of cells that are positive for a particular protein and the intensity of staining. This allows researchers to obtain a more comprehensive understanding of how much of a given protein is present in different tissues or under different conditions.

The IRS system uses a scale from 0 to 4 to rate the percentage of positive cells, with higher scores indicating greater levels of expression. Similarly, staining intensity is rated on a scale from 0 to 3, with higher scores indicating stronger staining.

By combining these two scores, researchers can generate an overall score that reflects both the amount and strength of protein expression in each sample. This information can be used to compare different tissues or experimental conditions and identify patterns or trends that may be relevant to disease processes or other biological phenomena.

### 2.5 Cell culture and treatment

The human CC line SW480 was procured from Icell Bioscience Biotechnology Co., Ltd. (Shanghai, China). All cell lines were maintained in Dulbecco's modified eagle medium (DMEM) supplemented with 10% fetal bovine serum (FBS), 100 U/mL penicillin, and 100 mg/mL streptomycin at 37°C with 5% CO_2_. To treat SW480 cells, 20 nM phorbol 12-myristate 13-acetate (PMA, Sigma, St Louis, USA) or Colivelin TFA (Proteintech, Wuhan, China) in DMEM medium for 12 h was used.

### 2.6 Clone formation assay

For the clone formation assay, 4  ×  10² SW480 cells were cultured in 2 mL of complete medium in 6-well plates for 7 days, with regular observations. The culture was terminated upon visible clone appearance. Cells were fixed with 2 mL of 4% paraformaldehyde for 10 min and stained with 2 mL of 5% crystal violet for 8 min. Petri dishes were inverted, and a transparent grid film was used to count clones visually, which were then recorded. The clone formation rate was calculated as follows: number of clones/number of inoculated cells × 100.

### 2.7 Transwell assays

Migration and invasion were assessed using a Boyden chamber assay (8-μm pore). CRC cells (1  ×  10^5^) were resuspended in 200 μL FBS-free medium and placed in the top chamber (BD, USA), while the lower chamber contained medium with 10% FBS. After 24 h, the cells were fixed, stained, and counted in six randomly selected fields under a microscope. The invasion assay was performed similarly using a modified Boyden chamber coated with Matrigel (BD Bioscience, USA).

### 2.8 Wound healing assay

Cells (1  ×  10^5^) were cultured in 6-well plates. After 16 h, the complete medium was replaced with low serum fresh medium (2%). Wounds were created using a 10 μL pipette tip once cells reached 90% confluence. The cells were washed twice with PBS to remove loose ones, and serum-free medium was added. To ensure comparability of wound areas, positioning marks were made at the center of each denuded surface. Scratch zones were photographed by inverted microscopy at 0 and 24 h. Image J software measured the migrating ability of cancer cells. Data presented were from three independent experiments.

### 2.9 RNA extraction and reverse transcription quantitative polymerase chain reaction (RT-qPCR) analysis

After killing the mice, their brains were quickly removed and dissected to isolate the cortex tissues. The tissues were then homogenized using a tissue grinder to break down cell membranes and release RNA. The Invitrogen TRIzol reagent (ThermoFisher) was used for RNA isolation, which involves adding chloroform to separate the aqueous phase containing RNA from other cellular components. After centrifugation, the RNA was collected from the upper layer and purified further.

To prepare cDNA for gene expression analysis, reverse transcription was performed using BeyoRT™ III cDNA transcriptase kit (Beyotime) according to the manufacturer’s instructions. This involved annealing primers specific to target genes with extracted RNA templates in a thermal cycler machine at appropriate temperatures and durations.

The sequences of these primers were carefully designed based on known gene sequences or predicted mRNA transcripts using bioinformatics tools such as BLAST or Primer3 software. They are critical for amplifying specific regions of interest during PCR (polymerase chain reaction) reactions that follow reverse transcription step. The sequences of these primers were as follows: TNF-α, 5′-AGGAGGGAGAACAGCAACTC-3′ (forward) and 5′-TGTATGAGAGGGACGGAACC-3′ (reverse); IL-1β, 5′-TCTCCAGTCAGGCTTCCTTG-3′ (forward) and 5′-ATCTGGACAGCCCAAGTCAA-3′(reverse); IL-6, 5′-CCACTGCCTTCCCTACTTCA-3′ (forward) and 5′-ACAGTGCATCATCGCTGTTC-3′ (reverse); Ki67, 5′-CAAAGCAGGAAGCAACAGGT-3′ (forward) and 5′-TGCTAATGTGCTCCTCCACA-3′ (reverse); E-cadherin, 5′-ATCGCCTACACCATCCTCAG-3′ (forward) and 5′-CAGCCTGAACCACCAGAGTA-3′ (reverse); N-cadherin, 5′-GGCAATCAAGTGGAGAACCC-3′ (forward) and 5′-CCCTCTGGAACAGACCCATT-3′ (reverse); vimentin, 5′-GAAAGTGTGGCTGCCAAGAA-3′ (forward) and 5′-GTTTGACTCCTGCTTTGCCT-3′ (reverse); GAPDH, 5′-GCCCTGACTGGAGATGAAGT-3′ (forward) and 5′-AAGCGACCCTTGGTGTCATA-3′ (reverse). Subsequently, RT-qPCR analyses were performed using the SYBR premix EX Taq™ (Takara, Kusatsu, Shiga, Japan) according to the manufacturer’s protocol in a Light Cycler 480 System (Roche Diagnostics, Grenzacherstrasse, Basel, Switzerland). The DNA amplification process is a crucial step in genetic research and analysis. In this study, the amplification was carried out using a specific protocol that involved maintaining the first cycle at 95°C for 30 s, followed by 40 cycles consisting of denaturation, annealing, and extension. The denaturation step involves separating the double-stranded DNA into single strands by heating it to high temperatures (95°C), while annealing allows primers to bind to complementary sequences on each strand of DNA. Extension then occurs when Taq polymerase adds nucleotides to the growing chain.

After amplification, gene expression levels were analyzed using the 2^−ΔΔCt^ method. This method involves comparing the expression level of an unknown sample against a calibrated sample with known gene expression levels. By calculating differences in threshold cycle values (Ct) between target genes and reference genes, researchers can determine relative changes in gene expression.

### 2.10 Western blot analysis

Protein extraction is a crucial step in many biological experiments, and the radioimmunoprecipitation assay lysis buffer (Beyotime, Shanghai, China) used in this study is a commonly used reagent for this purpose. The addition of 1 mM phenylmethylsulfonyl fluoride and protease/phosphatase inhibitors helps to prevent protein degradation during the extraction process. After solubilization, the proteins (30 µg) were separated by sodium dodecyl sulfate-polyacrylamide gel electrophoresis, which uses an electric field to separate proteins based on their size and charge. The separating gel has a higher concentration of acrylamide than the stacking gel, allowing for better resolution of smaller proteins. Finally, transferring gel to a PVDF membrane (Roche) allows for the detection of specific target proteins using antibodies. The following primary antibodies were used: anti-ki67, anti-E-cadherin, anti-N-cadherin, anti-vimentin, anti-p65, and anti-phospho-STAT3 (dilution of 1:800; Beyotime).

After washing, the membranes were carefully incubated with horseradish peroxidase-conjugated secondary antibodies (Pierce) to ensure optimal binding and detection of the target proteins. The use of a high-quality secondary antibody is crucial for obtaining accurate and reliable results in Western blot experiments. Following incubation, protein bands were visualized using an enhanced chemiluminescence Plus Western blot detection kit (Amersham Biosciences), which provides sensitive and consistent detection of even low-abundance proteins. The manufacturer’s instructions were followed precisely to ensure reproducibility across multiple experiments.

### 2.11 Data analysis

The one-way analysis of variance (ANOVA) is a statistical method used to compare the means of three or more groups. In this study, SPSS19.0 software was used to perform ANOVA on our data and calculate the mean ± S.D. (standard deviation). The results showed that there was a statistically significant difference between the groups with a two-sided value of *P* < 0.05, indicating that our hypothesis was supported by the data.

It is important to note that *P* < 0.01 was considered extremely statistically significant in this study, which suggests an even stronger level of evidence supporting our findings. This highlights the importance of using appropriate statistical methods and setting clear significance levels when analyzing research data.

## 3 Results

### 3.1 CEBPB affects the progression of UCCRC mice

**Figure 1:**
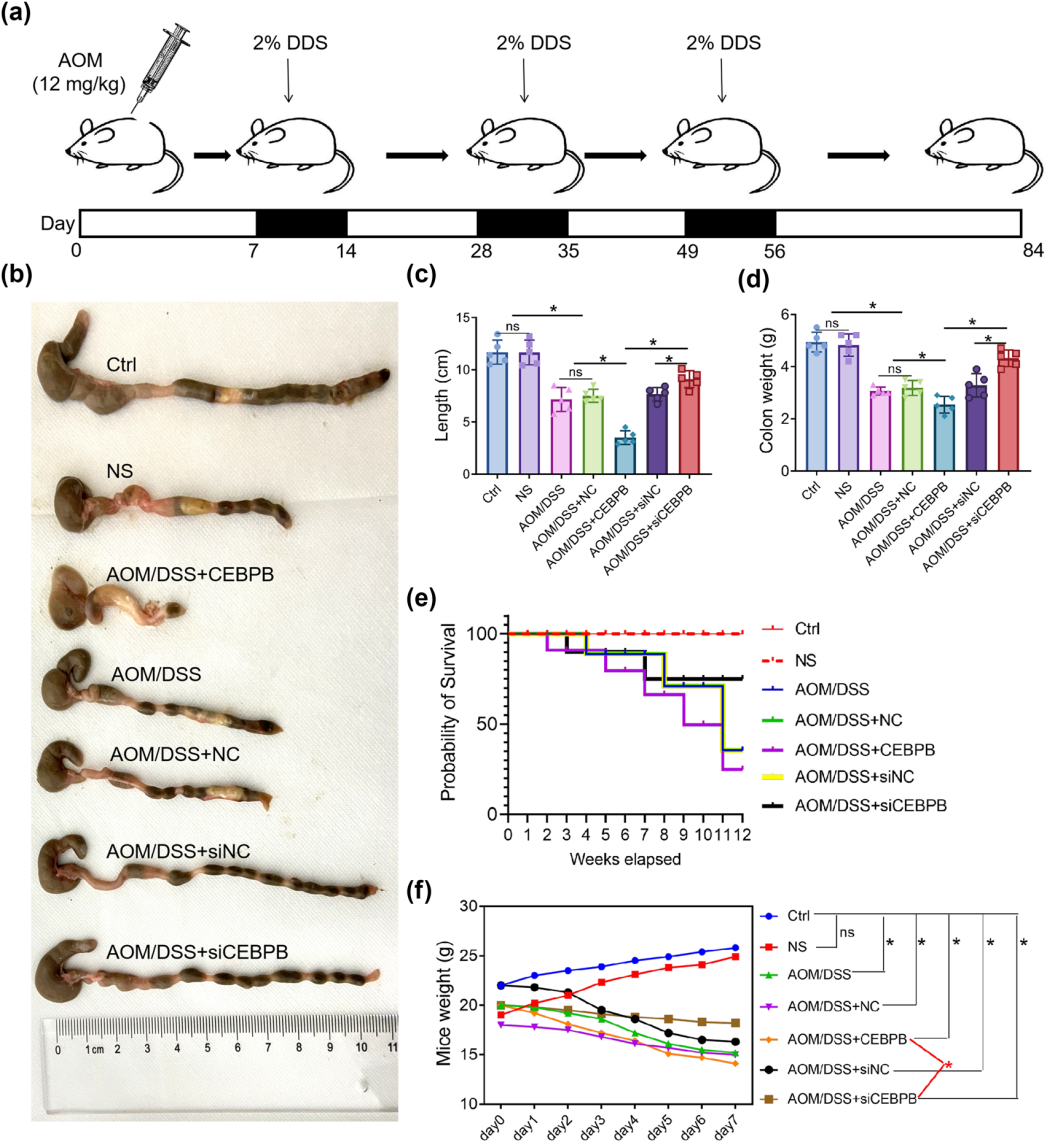
CEBPB affects the progression of UCCRC mice: (a) the modeling method of AOM/DSS-induced UCCRC in mice and treatment regimens, (b) representative photographs of AOM/DSS-induced UCCRC in mice, (c) colorectal length of mice, (d) colon weight of mice, (e) survival rate of mice, and (f) body weight of mice. *N* = 10, *P < 0.05.

**Figure 2:**
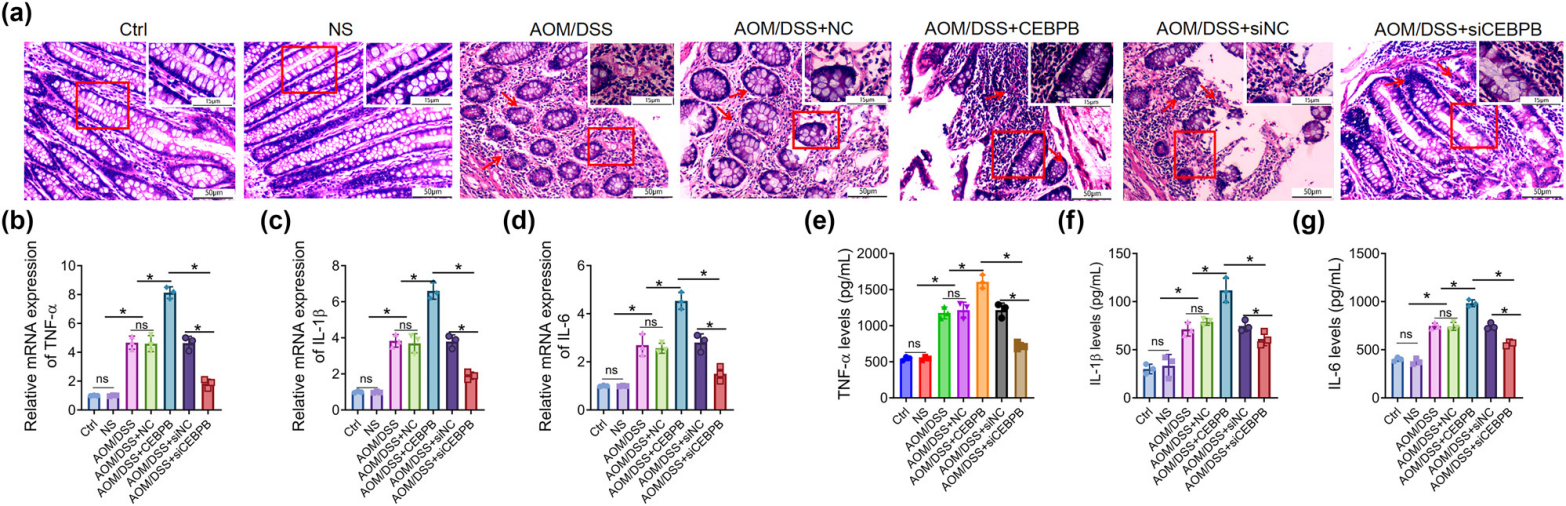
CEBPB affects the pathogenic alterations and inflammatory response in UCCRC mice, and representative images of H&E-stained colorectal tissues of mice. The scale bar is 200 μm (a); the concentrations of TNF-α (b), IL-6 (c), and IL-1β (d) in mice’s sera were determined with ELISA kits; the expression of TNF-α (e), IL-6 (f), and IL-1β (g) in colon tissues was determined with RT-qPCR assay, as per the manufacturer’s protocol. *P < 0.05. The red arrows represent the pathological changes.

To investigate the role of CEBPB in UCCRC, a mouse model was established using azoxymethane/dextran sodium sulfate (AOM/DSS) induction. The mice were meticulously selected and injected with AOM, followed by three cycles of DSS treatment as detailed in the Methods section and illustrated in Figure 1a. Subsequently, it was observed that the injection of CEBPB exacerbated the shortening of the colorectum and loss of colon weight induced by AOM/DSS (Figure 1b–d). Furthermore, there was a significant decrease in survival rates among UCCRC mice treated with CEBPB (Figure 1e). Concurrently, symptoms associated with UCCRC in these mice – including reduced activity, low energy levels, diminished responsiveness, and lacklusterness – were aggravated by CEBPB administration, as evidenced by decreased body weight (Figure 1d). Histological examination via HE staining revealed that CEBPB treatment led to more severe pathological alterations in UCCRC-affected mice (Figure 2a). Conversely, treatment with siCEBPB mitigated AOM/DSS-induced pathological changes in these animals. To further elucidate the influence of CEBPB on inflammatory responses within UCCRC mice, serum levels of pro-inflammatory factors such as TNF-α, IL-6, and IL-1β along with their expression within colon tissues were measured (Figure 2b–d). The results indicated that CEBPB significantly affected the levels of inflammatory cytokines associated with UCCRC progression. Specifically, it was found that CEBPB upregulated both serum concentrations and tissue expression levels of TNF-α, IL-6, and IL-1β, all critical mediators involved in inflammatory responses (Figure 2e–g).

These findings suggest that CEBPB may play a pivotal role in promoting inflammation within UCCRC, which could contribute to disease progression. Further studies are warranted to comprehensively understand the underlying mechanisms driving these effects.

CEBPB alters the expression of the related proteins and the activation of NF-κB/STAT3 signaling pathway.

**Figure 3:**
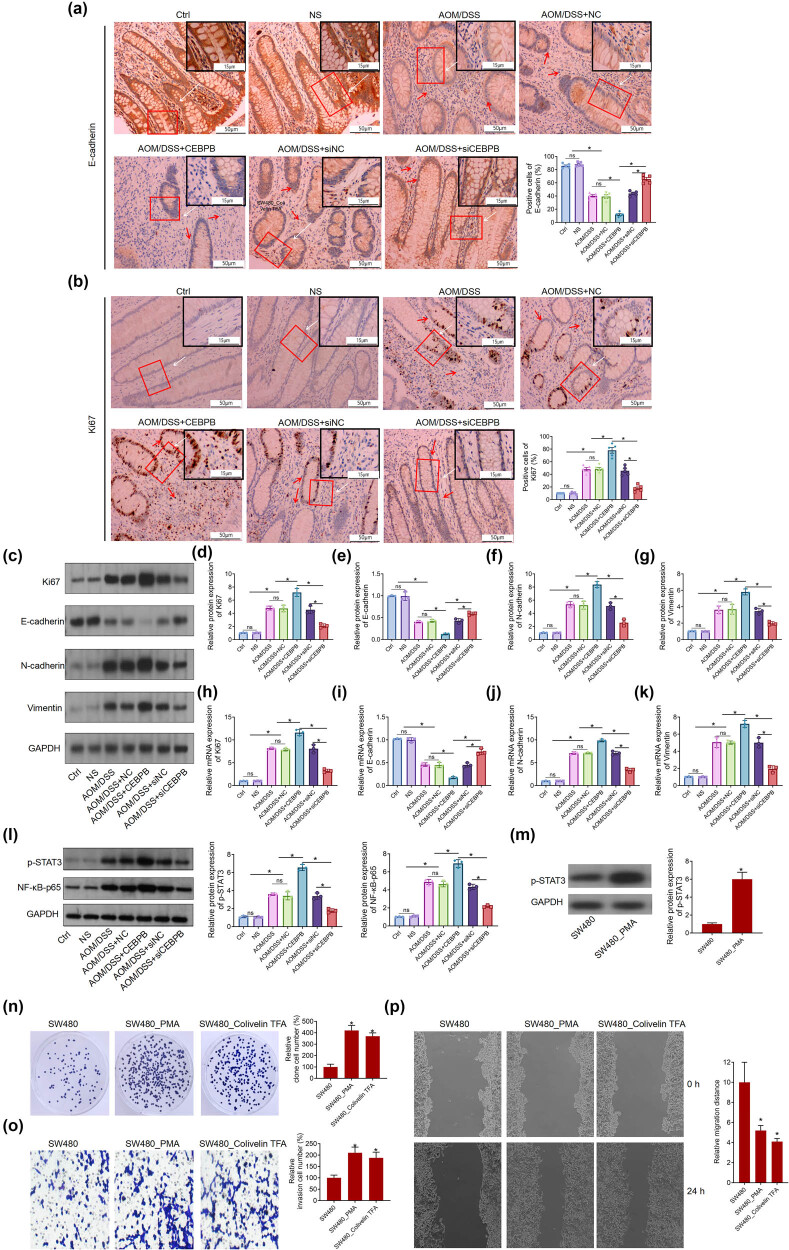
CEBPB alters the expression of the related proteins and the activation of NF-κB/STAT3 signaling pathway, IHC analysis of the protein expression of E-cadherin (a) and Ki67 (b) in the UCCRC tissues of mice. The scale bar is 50 μm and 100 μm. (c)–(g) Western blot analysis of the protein expression of Ki67, E-cadherin, N-cadherin, and vimtein in the UCCRC tissues of mice. RT-qPCR analysis of the mRNA expression of Ki67 (h), E-cadherin (i), N-cadherin (j), and vimentin (k) in UCCRC tissues of mice. (l) Western blot analysis of p-STAT3 and NF-κB-p65 expression UCCRC tissues of mice. (m) Western blot analysis of p-STAT3 expression in SW480 cells treatment with PMA. After PMA or Colivelin TFA treats SW480 cells, clone formation, transwell, and scratch-wound assay were used to detect cell proliferation (n), invasion (o), and migration (p). *N* = 3, *P < 0.05. PMA, phorbol 12-myristate 13-acetate; the red arrows represent the pathological changes.

Given that CEBPB accelerated the carcinogenesis of AOM/DSS-induced UCCRC in mice, we speculated whether CEBPB contributes to proliferation and metastasis in UCCRC. Intriguingly, IHC analysis revealed that AOM/DSS treatment increased Ki67 protein expression while decreasing E-cadherin protein levels in the colon tissues of mice (Figure 3a and b), which was consistent with the results obtained from Western blotting. Specifically, CEBPB led to a reduction in E-cadherin protein levels alongside an elevation of N-cadherin and vimentin protein levels in AOM/DSS-treated mice (Figure 3c–g). Furthermore, these findings were corroborated by RT-qPCR analysis (Figure 3h–k). Given that Ki67 is a pro-proliferation gene and E-cadherin, N-cadherin, and vimentin are associated with epithelial–mesenchymal transition (EMT), it can be inferred that CEBPB potentially induces proliferation and metastasis in UCCRC, thereby facilitating its progression.

Subsequently, significant activation of STAT3 phosphorylation and NF-κB-p65 expression in AOM/DSS-treated mice were observed. Notably, overexpression of CEBPB further activated the NF-κB/STAT3 signaling pathway within this context. Conversely, silencing CEBPB inhibited the activation of the NF-κB/STAT3 signaling pathway in AOM/DSS-treated mice (Figure 3l).

NF-κB enhances the phosphorylation of STAT3, thereby activating the NF-κB/STAT3 signaling pathway in colon cancer.

After treatment with NF-κB’s activator (PMA) on colon cell lines (SW480), there is a significant upregulation in the phosphorylation of STAT3 (Figure 3m). Furthermore, both PMA and colivelin TFA, an activator of STAT3, enhance proliferation, invasion, and migration in SW480 cells (Figure 3n–p).

CEBPB activates the NF-κB/STAT3 signaling pathway in UCCRC mice.

The NF-κB/STAT3 signaling pathway has been extensively investigated in relation to colon cancer, and it is widely acknowledged that this pathway plays a pivotal role in the pathogenesis of the disease. However, the precise mechanisms through which these transcription factors facilitate tumor progression remain inadequately understood. To illuminate this issue, researchers have focused their attention on CEBPB, a transcription factor that has been demonstrated to promote the advancement of colorectal cancer.

To investigate the influence of CEBPB on the activity of the NF-κB/STAT3 signaling pathway, researchers assessed its effects on two critical markers: NF-κB-p65 and STAT3. P65 serves as a crucial transcription factor within the NF-κB signaling cascade, while STAT3 is an essential component of the STAT3 signaling pathway. By analyzing alterations in the levels of these proteins following modifications to CEBPB expression or activity, researchers aim to elucidate how CEBPB facilitates tumor growth and metastasis.

**Figure 4:**
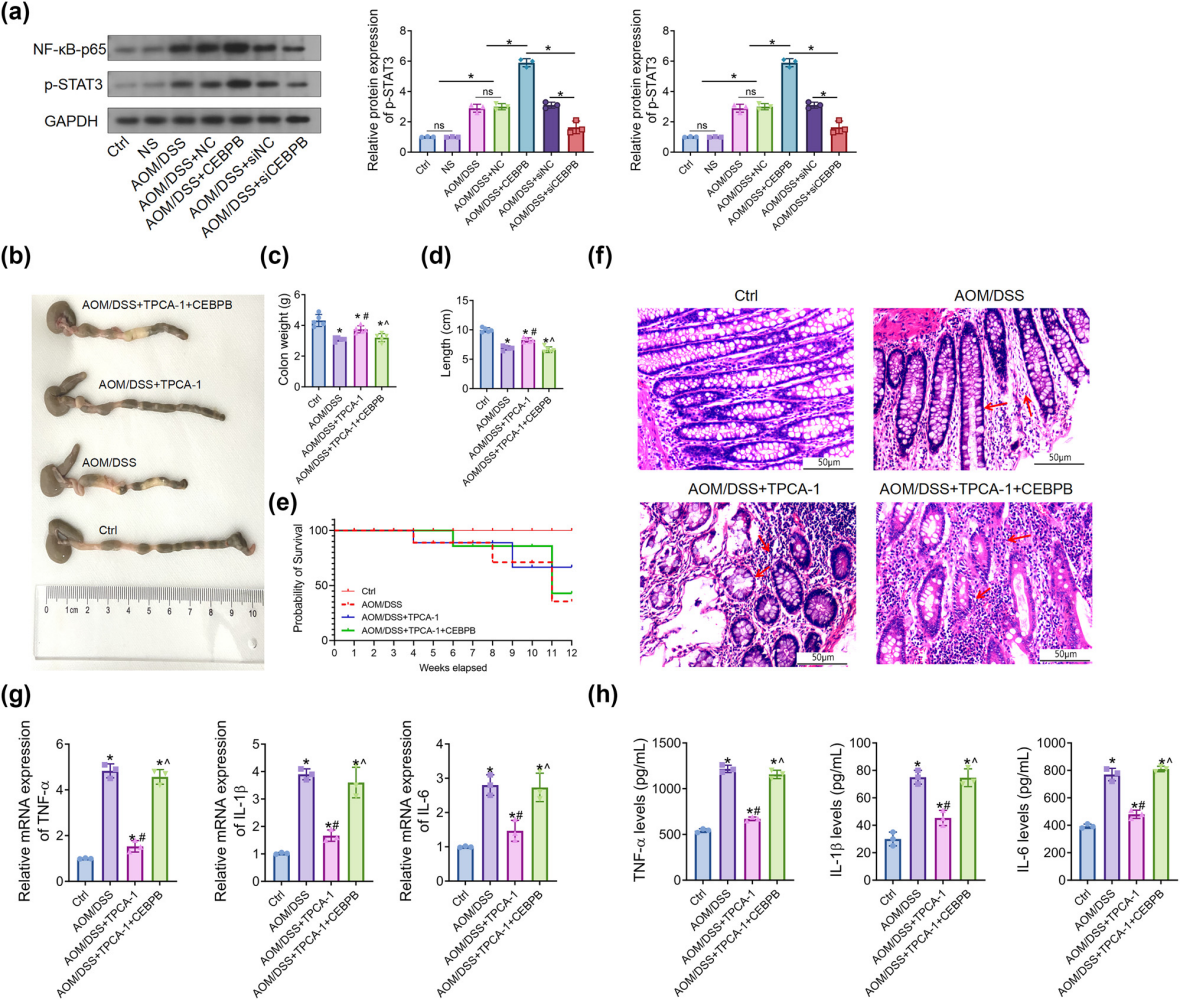
CEBPB activates the NF-κB/STAT3 signaling pathway in UCCRC mice: (a) Western blot analysis of the protein expression of NF-κB-p65 and p-STAT3 in the UCCRC tissues of mice. (b) Representative photographs of the different groups in mice and (c) the colon weight of mice, (d) colorectal length of mice, and (e) the survival rate of mice. (f) Representative images of H&E-stained colorectal tissues of mice. The scale bar is 200 μm. (g) The concentrations of TNF-α, IL-6, and IL-1β in mice’s sera were determined with ELISA kits. (h) The expression of TNF-α, IL-6, and IL-1β in colon tissues was determined with RT-qPCR assay, as per the manufacturer’s protocol. *P < 0.05. The red arrows represent the pathological changes.

It was revealed that the expression of the protein p65 and the phosphorylation level of STAT3 in colon tissues of UCCRC mice were significantly upregulated by CEBPB, as evidenced by Western blot analysis (Figure 4a). Furthermore, TPCA-1, a dual inhibitor of STAT3 and NF-κB signaling, was employed to inhibit the activation of NF-κB/STAT3 signaling in AOM/DSS-induced UCCRC mice. Our results indicated that CEBPB counteracted the effects of TPCA-1 on colon length; specifically, the colon length in the AOM/DSS group was markedly reduced compared to that observed in the normal control group. Following treatment with TPCA-1, there was a partial restoration of colon length; however, it remained lower than that seen in the normal control group. Notably, CEBPB transfection effectively negated the beneficial effects of TPCA-1 on improving colon length (Figure 4b–d). Additionally, TPCA-1 treatment enhanced survival rates among UCCRC mice, while CEBPB abolished this effect (Figure 4e).

**Figure 5:**
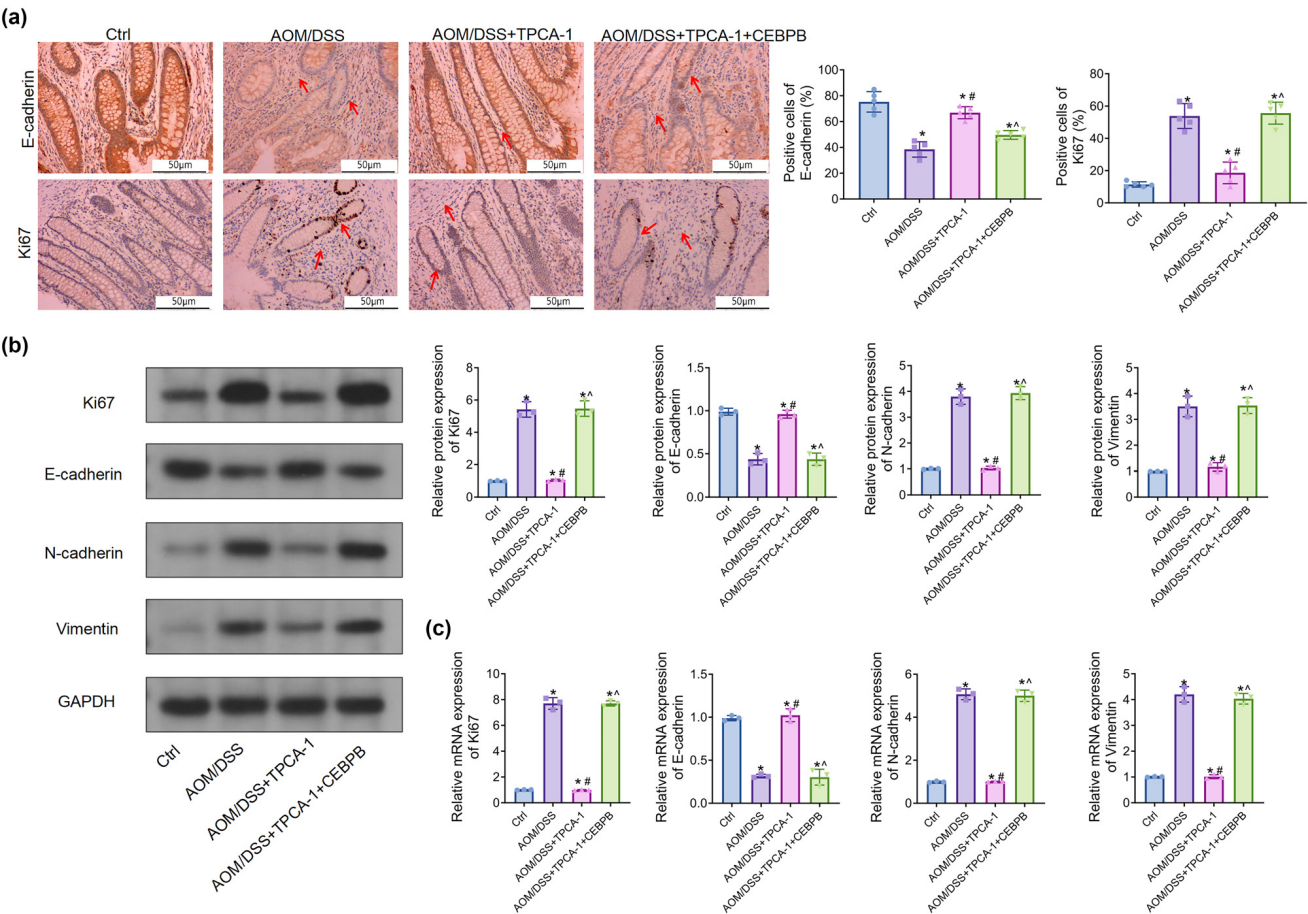
CEBPB regulates the expression of proteins via activating NF-κB/STAT3 signaling pathway, IHC analysis of the protein expression of E-cadherin and Ki67 in the UCCRC tissues of mice. The scale bar is 50 μm. (b) Western blot analysis of the protein expression of Ki67, E-cadherin, N-cadherin, and vimentin in the UCCRC tissues of mice. (c) Real-time RT-qPCR analysis of the mRNA expression of Ki67, E-cadherin, N-cadherin, and vimentin in colon tissues of mice. *N* = 3, *P < 0.05. The red arrows represent the pathological changes.

Subsequently, TPCA-1 ameliorated pathological changes in colon tissue induced by AOM/DSS; however, CEBPB inhibited these effects on colonic tissue within UCCRC mice (Figure 4f). It was found that CEBPB significantly elevated serum concentrations and expressions of TNF-α, IL-6, and IL-1β when compared to those in the AOM/DSS + TPCA-1 group (Figure 4g and h). Moreover, IHC and Western blot analyses demonstrated that TPCA-1 impeded protein expression levels associated with proliferation and EMT, including Ki67, N-cadherin, and vimentin (Figure 5a and b), while increasing E-cadherin expression levels. Importantly, CEBPB nullified these effects exerted by TPCA-1 in UCCRC mice.

Additionally, RT-qPCR analysis supported these findings regarding CEBPB’s influence on protein levels mentioned earlier (Figure 5c), demonstrating that CEPBP activated NF-kB/STAT3 signaling pathways within UCCRC mice.

## 4 Discussion

Intracellular inflammation is closely linked to colon cancer; however, the mechanisms underlying the transition from inflammation to malignancy remain to be fully elucidated [14]. Research has demonstrated that a critical state exists within the tumor microenvironment, specifically referred to as the tumor inflammatory microenvironment [15]. Previous research has indicated that inflammation-related factors may significantly contribute to the development of cancer metastases. As a crucial regulator of the inflammatory response, the nuclear transcription factors NF-κB and STAT3 play significant roles in modulating this process [16]. A detrimental cycle of “inflammation-tumor” is present within the tumor microenvironment. Once a tumor is established in the human body, the inflammatory environment created by tumor cells can activate the NF-κB and STAT3 signaling pathways [17]. This activation leads to an increased expression of various inflammatory cytokines within the tumor microenvironment, including TNF-α, IL-6, IL-1β, and other cytokines. The activation of the NF-κB signaling pathway enhances the secretion of the cytokine IL-6 [18]. Furthermore, *in vivo* expression of tumor suppressor genes P53 and P65 is influenced by IL-6 secretion, which subsequently induces cellular carcinogenesis and facilitates the differentiation of tumor cells [19]. Additionally, TNF-α secretion can also promote the occurrence of inflammatory responses *in vivo*. The secretion of the cytokine IL-6 can also activate the STAT3 signaling pathway, thereby influencing the entire process of tumor initiation and progression [20].

It has been reported that the activation of the NF-κB signaling pathway can stimulate the release of inflammatory factors, thereby triggering an inflammatory response that enhances the immune response [21]. NF-κB plays a crucial role in conveying information by modulating tissue repair processes and engaging in tumor-associated inflammation. This involvement ultimately contributes to the maintenance of tumor growth and invasion. It has been demonstrated that the STAT3 signaling pathway significantly influences tumor cell growth, reproduction, metastasis, and apoptosis [22,23]. Furthermore, it plays a crucial role in tumor formation and development. It have demonstrated that CEBPB functions as a transcription factor, potentially playing a significant role in the inflammatory state [24]. Blocking CEBPB using siRNA significantly diminishes the production of IL-6 and IL-8 induced by tunicamycin. Endoplasmic reticulum stress can modulate the inflammatory response and extracellular matrix degradation through the activation of CEBPB expression. STAT3, CEBPB, and NF-κB are recognized as significant biomarkers in the pathogenesis of psoriasis and may serve as potential drug targets for its treatment [25]. Further research has established a connection between CEBPB and the enhanced nuclear expression of phosphorylated STAT3 [26]. Consequently, CEBPB may play a pivotal role in the “inflammation-cancer” nexus by modulating the NF-κB/STAT3 signaling pathway. In this study, CEBPB was utilized as a focal point to explore the mechanisms underlying UCCRC development.

In the present study, CEBPB was found to enhance the secretion and expression of inflammatory cytokines (TNF-α, IL-6, and IL-1β), exacerbate AOM/DSS-induced pathological loss of colon tissue, and upregulate the expression of oncogenes. The inhibition of CEBPB mitigated the inflammation-driven carcinogenic effects associated with AOM/DSS, indicating that CEBPB facilitates the progression from colitis to colon cancer. Subsequently, we observed that CEBPB increased the expression levels of markers related to the NF-κB/STAT3 signaling pathway (p65 and STAT3) induced by AOM/DSS. Treatment with a CEBPB blocker in AOM/DSS mice significantly reduced p65 and STAT3 expression. These findings suggest that CEBPB may promote the activation of NF-κB/STAT3 signaling.

To further validate this observation, an NF-κB/STAT3 signaling inhibitor (TPCA-1) was administered to AOM/DSS mice in order to assess both inflammatory responses and pathological damage within the colon tissue. Our results indicated that CEBPB obstructed TPCA-1’s therapeutic efficacy on UCCRC. Notably, our data demonstrated the activation of the NF-κB/STAT3 pathway in UCCRC mice alongside evidence that activated NF-κB stimulated STAT3 signaling. The activated NF-κB/STAT3 pathway was shown to facilitate the proliferation, invasion, and migration of colon cells. This underscores the significant role played by the NF-κB/STAT3 pathway in UCCRC development while highlighting how NF-κB can regulate STAT3 activation.

This study has validated the role of CEBPB in UCCRC mice at the animal level; however, the transition from colitis to colorectal cancer is a protracted process that involves epithelial phenotype switching. Future investigations will concentrate on the transformation of colon epithelial cells and tumor cells to gain a deeper understanding of how CEBPB functions in UCCRC.

Collectively, our findings indicate that CEBPB exacerbates pathological tissue damage in the colon and activates inflammatory responses. NF-κB enhances the phosphorylation levels of STAT3. The NF-κB/STAT3 pathway is activated in the colon tissues of UCCRC mice. This activation promotes the proliferation, invasion, and migration of colon cancer cells. In summary, our results suggest that CEBPB facilitates the progression of UCCRC by activating the NF-κB/STAT3 signaling cascade, thereby contributing to a better understanding of the mechanisms underlying the progression from colitis to colon cancer.

## References

[1] Le Berre C, Honap S, Peyrin-Biroulet L. Ulcerative colitis. Lancet. 2023;402(10401):571–84. 10.1016/S0140-6736(23)00966-2 37573077

[2] Kaenkumchorn T, Wahbeh G. Ulcerative colitis: making the diagnosis. Gastroenterol Clin North Am. 2020;49(4):655–69. Epub 2020/10/31. 10.1016/j.gtc.2020.07.001 33121687

[3] Segal JP, LeBlanc JF, Hart AL. Ulcerative colitis: an update. Clin Med. 2021;21(2):135–9. Epub 2021/03/26. 10.7861/clinmed.2021-0080 PMC8002778 33762374

[4] Elhefnawy EA, Zaki HF, El Maraghy NN, Ahmed KA, Abd El-Haleim EA. Genistein and/or sulfasalazine ameliorate acetic acid-induced ulcerative colitis in rats via modulating INF-γ/JAK1/STAT1/IRF-1, TLR-4/NF-κB/IL-6, and JAK2/STAT3/COX-2 crosstalk. Biochem Pharmacol. 2023;214:115673. Epub 2023/07/07. 10.1016/j.bcp.2023.115673 37414101

[5] Zhang N, Liu C, Jin L, Zhang R, Wang T, Wang Q, et al. Ketogenic diet elicits antitumor properties through inducing oxidative stress, inhibiting MMP-9 expression, and rebalancing M1/M2 tumor-associated macrophage phenotype in a mouse model of colon cancer. J Agric Food Chem. 2020;68(40):11182–96. Epub 2020/08/14. 10.1021/acs.jafc.0c04041 32786841

[6] Li B, Du P, Du Y, Zhao D, Cai Y, Yang Q, et al. Luteolin alleviates inflammation and modulates gut microbiota in ulcerative colitis rats. Life Sci. 2021;269:119008. Epub 2021/01/13. 10.1016/j.lfs.2020.119008 33434535

[7] Liuu S, Nepelska M, Pfister H, Gamelas Magalhaes J, Chevalier G, Strozzi F, et al. Identification of a muropeptide precursor transporter from gut microbiota and its role in preventing intestinal inflammation. Proc Natl Acad Sci U S A. 2023;120(52):e2306863120. Epub 2023/12/21. 10.1073/pnas.2306863120 PMC10756304 38127978

[8] He W, Tang M, Gu R, Wu X, Mu X, Nie X. The role of p53 in regulating chronic inflammation and panoptosis in diabetic wounds. Aging Dis. 2024;16(1):373–93. Epub 2024/02/20. 10.14336/AD.2024.0212 PMC11745441 38377027

[9] Watanabe S, Hibiya S, Katsukura N, Kitagawa S, Sato A, Okamoto R, et al. Influence of chronic inflammation on the malignant phenotypes and the plasticity of colorectal cancer cells. Biochem Biophys Rep. 2021;26:101031. Epub 2021/06/08. 10.1016/j.bbrep.2021.101031 PMC8167241 34095556

[10] Kim SL, Shin MW, Seo SY, Kim SW. Lipocalin 2 potentially contributes to tumorigenesis from colitis via IL-6/STAT3/NF-κB signaling pathway. Biosci Rep. 2022;42(5):BSR20212418. Epub 2022/04/27. 10.1042/BSR20212418 PMC9109459 35470375

[11] Zhou Z, Shu Y, Bao H, Han S, Liu Z, Zhao N, et al. Stress-induced epinephrine promotes epithelial-to-mesenchymal transition and stemness of CRC through the CEBPB/TRIM2/P53 axis. J Transl Med. 2022;20(1):262. Epub 2022/06/08. 10.1186/s12967-022-03467-8 PMC9172202 35672760

[12] Ge CY, Wei LY, Tian Y, Wang HH. A seven-NF-κB-related gene signature may distinguish patients with ulcerative colitis-associated colorectal carcinoma. Pharmgenomics Pers Med. 2020;13:707–18. Epub 2020/12/11. 10.2147/PGPM.S274258 PMC7719442 33299340

[13] Horváth G, Horváth A, Reichert G, Böszörményi A, Sipos K, Pandur E. Three chemotypes of thyme (Thymus vulgaris L.) essential oil and their main compounds affect differently the IL-6 and TNFα cytokine secretions of BV-2 microglia by modulating the NF-κB and C/EBPβ signalling pathways. BMC Complement Med Ther. 2021;21(1):148. Epub 2021/05/24. 10.1186/s12906-021-03319-w PMC8140451 34022882

[14] Parker BJ, Wearsch PA, Veloo ACM, Rodriguez-Palacios A. The genus alistipes: gut bacteria with emerging implications to inflammation, cancer, and mental health. Front Immunol. 2020;11:906. Epub 2020/06/26. 10.3389/fimmu.2020.00906 PMC7296073 32582143

[15] Tomkins-Netzer O, Niederer R, Greenwood J, Fabian ID, Serlin Y, Friedman A, et al. Mechanisms of blood-retinal barrier disruption related to intraocular inflammation and malignancy. Prog Retinal Eye Res. 2024;99:101245. Epub 2024/01/20. 10.1016/j.preteyeres.2024.101245 38242492

[16] Malki A, ElRuz RA, Gupta I, Allouch A, Vranic S, Al Moustafa AE. Molecular mechanisms of colon cancer progression and metastasis: recent insights and advancements. Int J Mol Sci. 2020;22(1):130. Epub 2020/12/31. 10.3390/ijms22010130 PMC7794761 33374459

[17] Zhang Z, Du J, Xu Q, Xing C, Li Y, Zhou S, et al. Adiponectin suppresses metastasis of nasopharyngeal carcinoma through blocking the activation of NF-κB and STAT3 signaling. Int J Mol Sci. 2022;23(21):12729. Epub 2022/11/12. 10.3390/ijms232112729 PMC9658954 36361525

[18] Zhang Y, Wang S, Dai X, Liu T, Liu Y, Shi H, et al. Simiao San alleviates hyperuricemia and kidney inflammation by inhibiting NLRP3 inflammasome and JAK2/STAT3 signaling in hyperuricemia mice. J Ethnopharmacol. 2023;312:116530. Epub 2023/04/26. 10.1016/j.jep.2023.116530 37098372

[19] Hirano T. IL-6 in inflammation, autoimmunity and cancer. Int Immunol. 2021;33(3):127–48. Epub 2020/12/19. 10.1093/intimm/dxaa078 PMC7799025 33337480

[20] Ren Q, Tao S, Guo F, Wang B, Yang L, Ma L, et al. Natural flavonol fisetin attenuated hyperuricemic nephropathy via inhibiting IL-6/JAK2/STAT3 and TGF-β/SMAD3 signaling. Phytomedicine. 2021;87:153552. Epub 2021/05/18. 10.1016/j.phymed.2021.153552 33994251

[21] Mukherjee T, Kumar N, Chawla M, Philpott DJ, Basak S. The NF-κB signaling system in the immunopathogenesis of inflammatory bowel disease. Sci Signal. 2024;17(818):eadh1641. Epub 2024/01/09. 10.1126/scisignal.adh1641 38194476

[22] Ouyang S, Li H, Lou L, Huang Q, Zhang Z, Mo J, et al. Inhibition of STAT3-ferroptosis negative regulatory axis suppresses tumor growth and alleviates chemoresistance in gastric cancer. Redox Biol. 2022;52:102317. Epub 2022/04/29. 10.1016/j.redox.2022.102317 PMC9108091 35483272

[23] Zou S, Tong Q, Liu B, Huang W, Tian Y, Fu X. Targeting STAT3 in cancer immunotherapy. Mol Cancer. 2020;19(1):145. Epub 2020/09/26. 10.1186/s12943-020-01258-7 PMC7513516 32972405

[24] Westin ER, Khodadadi-Jamayran A, Pham LK, Tung ML, Goldman FD. CRISPR screen identifies CEBPB as contributor to dyskeratosis congenita fibroblast senescence via augmented inflammatory gene response. G3 (Bethesda). 2023;13(11):jkad207. Epub 2023/09/17. 10.1093/g3journal/jkad207 PMC10627266 37717172

[25] Berendsen S, van Bodegraven E, Seute T, Spliet WGM, Geurts M, Hendrikse J, et al. Adverse prognosis of glioblastoma contacting the subventricular zone: Biological correlates. PLoS One. 2019;14(10):e0222717. Epub 2019/10/12. 10.1371/journal.pone.0222717 PMC6788733 31603915

[26] Han JH, Jang KW, Myung CS. Garcinia cambogia attenuates adipogenesis by affecting CEBPB and SQSTM1/p62-mediated selective autophagic degradation of KLF3 through RPS6KA1 and STAT3 suppression. Autophagy. 2022;18(3):518–39. Epub 2021/06/09. 10.1080/15548627.2021.1936356 PMC9037513 34101546

